# Increased serum phenylalanine/tyrosine ratio associated with the psychiatric symptom of anti-NMDAR encephalitis

**DOI:** 10.3389/fneur.2024.1434139

**Published:** 2024-10-09

**Authors:** Jia Ma, Zhidong Zheng, Jiali Sun, Huabing Wang, Hengri Cong, Yuzhen Wei, Yuetao Ma, Kai Feng, Linlin Yin, Xinghu Zhang

**Affiliations:** ^1^Department of Neuroinfection and Neuroimmunology, Beijing Tiantan Hospital, Capital Medical University, Beijing, China; ^2^Department of Neurology, Beijing Shunyi Hospital, Beijing Shunyi Teaching Hospital of Capital Medical University, Beijing, China; ^3^China National Clinical Research Center for Neurological Diseases, Beijing Tiantan Hospital, Capital Medical University, Beijing, China

**Keywords:** anti-NMDAR encephalitis, phenylalanine, tyrosine, aromatic amino acids, psychiatric syndrome, serum Phe/Tyr ratio

## Abstract

**Background:**

Encephalitis associated with antibodies against the *N*-methyl-*D*-aspartate receptor (NMDAR) results in a distinctive neuro-psychiatric syndrome. It has been reported that the serum phenylalanine-tyrosine (Phe/Tyr) ratio increases during infection. However, the connection between phenylalanine-tyrosine metabolism and psychiatric symptoms remains unclear.

**Methods:**

We enrolled 24 individuals with anti-NMDAR encephalitis and 18 individuals with non-inflammatory neurological diseases (OND). Chromatography was used to measure serum levels of phenylalanine and tyrosine. Serum and cerebrospinal fluid (CSF) TNF-*α* levels were obtained from the clinical database. The modified Rankin Scale (mRS) score and Glasgow Coma Scale (GCS) score were recorded during the acute phase. The area under the curve (AUC) of the receiver operating characteristic curve was used to assess prediction efficacy.

**Results:**

In NMDAR patients, levels of serum Phe and the ratio of serum Phe/Tyr were higher compared to OND patients. The serum Phe/Tyr ratio was also elevated in NMDAR patients with psychiatric syndrome. Furthermore, serum Phe and Tyr levels were correlated with inflammatory indexes.

**Conclusion:**

The serum Phe/Tyr ratio is elevated in NMDAR patients with psychiatric syndrome and is associated with severity. Therefore, the serum Phe/Tyr ratio may serve as a potential prognostic biomarker.

## 1 Introduction

Anti-*N*-methyl-*D*-aspartate receptor (NMDAR) encephalitis is a rare immune-mediated disease of the central nervous system, formally recognized in 2007, with an estimated prevalence of 1.5 per million population per year (1). NMDAR encephalitis is characterized by a complex neuropsychiatric clinical profile and the presence of cerebrospinal fluid (CSF) antibodies against the GluN1 subunit of the NMDAR (2). The pathogenesis involves autoantibody targeting of NMDAR proteins on the cell surface, resulting in glutamatergic synaptic hypofunction, paraneoplastic autoimmunity, and viral infection (such as herpes simplex virus) (3). However, the precise mechanism initiating this antibody response, as well as the roles of tumors, infectious triggers, and immune reactivation in anti-NMDAR encephalitis, still requires investigation.

Anti-NMDAR encephalitis exhibits a female predominance, age distribution (median 21 years, range < 1–85 years), frequency of tumor associations (mostly ovarian teratoma), and a multistage symptom progression (beginning with a viral prodrome; followed by prominent psychiatric symptoms, agitation, and confusion; progressing to severe neurological features like seizures, movement abnormalities, autonomic instability, or hypoventilation) (1, 4–6). In the initial stages of this disease, 90% of patients exhibit prominent psychiatric or behavioral symptoms, making differentiation from primary psychiatric diseases challenging (1, 7). Additionally, the neuropsychiatric symptoms of post-acute anti-NMDAR encephalitis resemble those observed in schizophrenia (8). However, NMDAR encephalitis patients with isolated psychiatric episodes can respond to immunotherapy, whereas schizophrenia rarely improves with immunotherapy (7). Thus, identifying biomarkers for early screening in clinical practice is crucial for timely and effective interventions in anti-NMDAR encephalitis patients.

Existing research on biomarkers for anti-NMDAR encephalitis primarily focuses on neuroinflammatory proteins, such as soluble signaling proteins utilized by immune cells to regulate inflammatory responses (9–11). Additionally, several studies have explored the innate immune system and axonal damage in relation to this disorder (12–14). Notably, there is limited research concerning the association between metabolism, psychiatric symptoms, and anti-NMDAR encephalitis.

Preliminary studies suggest that metabolic alterations are present in many neurological disorders, including Alzheimer’s and Parkinson’s diseases, as well as other autoimmune disorders (15–18). Fitzgerald et al. identified shifts in aromatic amino acid metabolism in multiple sclerosis patients (19). Singh et al. found a distinct metabolic profile of disease state in a chronic mouse model of multiple sclerosis (20). In the treatment of psychiatric disorders, Fukuwatari et al. discovered the potential of amino acid treatment through the regulation of tryptophan production (21). Furthermore, Tohru et al. concluded that peripheral blood levels of endogenous glycine and alanine could serve as symptomatic markers in schizophrenia (22). These findings highlight the potential application of amino acid metabolism in the treatment and prevention of neurological disorders.

## 2 Methods

### 2.1 Study design, participants, and sample collection

This study enrolled 24 patients with anti-NMDAR encephalitis and 18 patients with non-inflammatory neurological disorders from the Neuroinfection and Neuroimmunology Center, Department of Neurology, Beijing Tiantan Hospital, between January 2017 and September 2021. The anti-NMDAR encephalitis patients were diagnosed based on the revised criteria published in 2016, which consider six major symptom groups and laboratory results, excluding other disorders (23). The non-inflammatory neurological disorders (OND) group (*n* = 18) served as the normative data (negative control). These patients had neurological conditions without central nervous system autoimmune antibodies and were non-inflammatory in the central nervous system. The OND group included patients with benign intracranial hypertension (*n* = 10), peripheral neuropathy (*n* = 2), diabetic retinopathy (*n* = 1), hypotensive headache (*n* = 1), anxiety disorders (*n* = 3), and hypertension (*n* = 1). None of the OND group individuals tested positive for serum or CSF IgG antibodies against the GluN1 subunit of the NMDA receptor.

All CSF and serum samples were collected from patients during the acute phase of anti-NMDAR encephalitis and before treatment. This study was conducted in compliance with the ethical principles of the Declaration of Helsinki. The experimental protocols were approved by the Ethics Committee of Beijing Tiantan Hospital, Capital Medical University (No. KY2015-031-02). All participants (or their legal representatives) were informed about the study and provided written informed consent before participation.

### 2.2 Clinical parameters

The study’s clinical parameters included demographic information (age and gender), the modified Rankin Scale (mRS), and the Glasgow Coma Scale (GCS). The functional outcomes of patients were assessed using the GCS and mRS, with data retrieved from digital medical records for analysis.

The modified Rankin Scale, a 7-level scale, measures global disability. The 7 levels range from 0 to 6, representing normal health without symptoms (0), symptomatic but nondisabled (1), disabled but independent (2), dependent but ambulatory (3), non-ambulatory nor capable of self-care (4), requiring constant care (5), and death (6) (24). In this study, mRS assessments were conducted through face-to-face structured interviews. The severity of acute phase neurological impairments was categorized as a good outcome (moderate, scores ≤3) or poor outcome (severe, scores >3) based on mRS scores.

The Glasgow Coma Scale (GCS) provides a structured method for assessing the level of consciousness and estimating long-term prognosis. The GCS is scored based on responses in three subscales: eye-opening (E, score range 1–4), verbal (V, score range 1–5), and motor (M, score range 1–6), with the total score ranging from 3 to 15. The total score reflects the severity of neurological injury: scores of 3–8 indicate severe injury, 9–12 indicate moderate injury, and 13–15 indicate mild injury (25).

A comprehensive dataset was gathered using standard medical procedures to measure tumor necrosis factor-*α* (TNFα).

### 2.3 Sample preparation

Blood samples were collected in the yellow-top (with coagulant accelerator and separation gel) blood collection tubes. Blood samples were centrifuged at 1200 g for 10 min at room temperature to collect the serum. CSF samples were centrifuged at 1000 g for 10 min at room temperature. The supernatant from both serum and CSF samples was carefully collected, divided into microtubes, and stored at-80°C for subsequent analysis. Following freezing, the serum and CSF samples were thawed at 4°C. To 100 μL of serum or CSF sample, 300 μL of a methanol mixture containing the internal standards d8-phenylalanine and d2-tyrosine was added. Both d8-phenylalanine and d2-tyrosine were dissolved in methanol. The organic phase in the mobile phase was methanol. Methanol was therefore used as a solvent for d8-phenylalanine and d2-tyrosine. The mixture was stirred for 1 min and then centrifuged at 20,000 g for 15 min at 4°C. The supernatant was carefully separated, placed in a liquid phase vial, and prepared for testing. Subsequently, 1 μL of 10% formic acid was added in water (pH = 1.4) to the supernatant, mixed thoroughly, and then for sample loading.

### 2.4 Measurement of serum phenylalanine and tyrosine

The liquid chromatography–tandem mass spectrometry (LC–MS) methods used in this study to quantify serum phenylalanine and tyrosine have been previously described (26, 27). The performance parameters of our method are comparable to those reported in earlier studies. The multiple reaction monitoring (MRM) parameters for both the analytes and internal standards are presented in Supplementary Table S1. The mass spectrometry procedures and parameters utilized in this study are detailed below. The LC–MS system utilized includes a SCIEX QTRAP 6500+ mass spectrometer (SCIEX, Foster City, CA, United States), which is directly interfaced with a high-performance liquid chromatography (HPLC) system equipped with a binary pump and an autosampler (Shimadzu Scientific Instruments, Inc., Columbia, MD, United States). This system operates in positive electrospray ionization (ESI) mode with a capillary voltage of 5.5 kV, desolations maintained at 550°C, and chromatographic separation occurring on a Phenomenex Kinetex 2.6 μm EVO C18 100 Å column (2.1 × 100 mm, 2.6 μm). The column temperature is set at 40°C. The gradient elution was programmed with the mobile phase consisting of A: 0.1% formic acid (FA, Optima, Fisher A117-50) in water, B: 0.1% FA in acetonitrile (ACN, Optima, Fisher A955-212). The flow rate of 0.7 mL/min is as follows: 0–2 min, 30–70% B (v/v); 2–4.5 min, 70–80% B (v/v); 4.5–6 min, 80–95% B (v/v); 6–6.2 min, 95–30% B (v/v). We used Waters amino acid mixed standard solution (WAT088122) and SIGMA internal standard mixture (96378-1ML) as the quantitative internal standards. The system runs in this state until the end of 7 min. The autosampler temperature was set at 4°C, and the injection volume was 1 μL. Data acquisition and processing were performed using Analyst software version 1.7.3 (SCIEX) and MultiQuant 3.2 (SCIEX).

### 2.5 Statistical analysis

All statistical analyses were conducted using SPSS version 29.0 (IBM, Armonk, NY, US) and GraphPad Prism version 9.5 (GraphPad Software Inc., US). Continuous variables were presented as the mean and standard deviation (SD) for normally distributed data. The Student’s *t*-test was used to analyze differences between two groups for parametric tests. The Kruskal-Wallis test was applied for multiple comparisons for non-parametric tests. Correlation coefficients between serum Phe/Tyr ratio, serum phenylalanine levels, serum tyrosine levels, mRS, GCS, serum TNF-*α* levels, and CSF TNF-α levels were calculated using nonparametric methods. A *p*-value <0.05 was considered statistically significant. The performance of the discriminant was assessed by measuring the area under the curve (AUC) of the receiver operating characteristic (ROC) curve. The G*power software was applied to calculate the statistical power in our study. The area under the ROC curve is a widely used metric for evaluating the accuracy and discriminatory power of prediction models (28).

## 3 Results

### 3.1 Patient characteristics

The clinical signs and baseline biomarker levels of individuals with anti-NMDAR encephalitis (*n* = 24, 12 males and 12 females) and OND (*n* = 18, 9 males and 9 females) are presented in Table 1. The diagnosis of anti-NMDAR encephalitis was confirmed by two doctors. No significant differences in gender or age were found between the anti-NMDAR encephalitis group and the control group. All OND controls tested negative for specific CSF antibodies. The mRS scale was used to measure the degree of disability or life dependence, and the GCS score was used to assess consciousness levels.

**Table 1:** Clinical and demographic characteristics of all participants.

Subject details	OND (*n* = 18)	anti-NMDAR encephalitis (*n* = 24)
Age, mean ± SD, y	38.06 ± 6.30	32.92 ± 13.05
Sex, No. (%)		
Male	9 (50%)	12 (50%)
Female	9 (50%)	12 (50%)
modified Rankin Scale (mRS) score		
Score 0, no symptoms	–	3 (12.5%)
Score 1, nondisabling symptoms	–	10 (41.7%)
Score 2, minor symptoms	–	1 (4.1%)
Score 3, moderate symptoms	–	4 (16.7%)
Score 4, moderately severe symptoms	–	3 (12.5%)
Score 5, severely disabled	–	3 (12.5%)
Score 6, dead	–	0
Glasgow Coma Scale (GCS)		
GCS 13–15, mild head injury	–	16 (66.7%)
GCS 9–12, moderate head injury	–	3 (12.5%)
GCS 3–8, severe head injury	–	5 (20.8%)
serum Phe levels (μmol/L), mean ± SD	58.86 ± 10.49	74.73 ± 20.85^*^
serum Tyr levels (μmol/L), mean ± SD	52.95 ± 21.81	53.76 ± 13.13
serum Phe/Tyr ratio, mean ± SD	1.12 ± 0.34	1.43 ± 0.38^*^
CSF TNF-𝛼, mean ± SD	–	10.14 ± 8.75
CSF Phe levels (μmol/L), mean ± SD	7.94 ± 3.59	8.38 ± 2.09
CSF Tyr levels (μmol/L), mean ± SD	7.01 ± 2.48	6.69 ± 1.24

All statistical analyses were assessed by student *t*-test. *p < 0.05. SD, standard deviation; y, year; CSF, cerebrospinal fluid; mRS, modified Rankin Scale; GCS, Glasgow Coma Scale; OND, non-inflammatory neurological diseases.

### 3.2 Serum Phe/Tyr ratio elevated in anti-NMDAR encephalitis and associated with psychiatric syndrome

**Figure 1:**
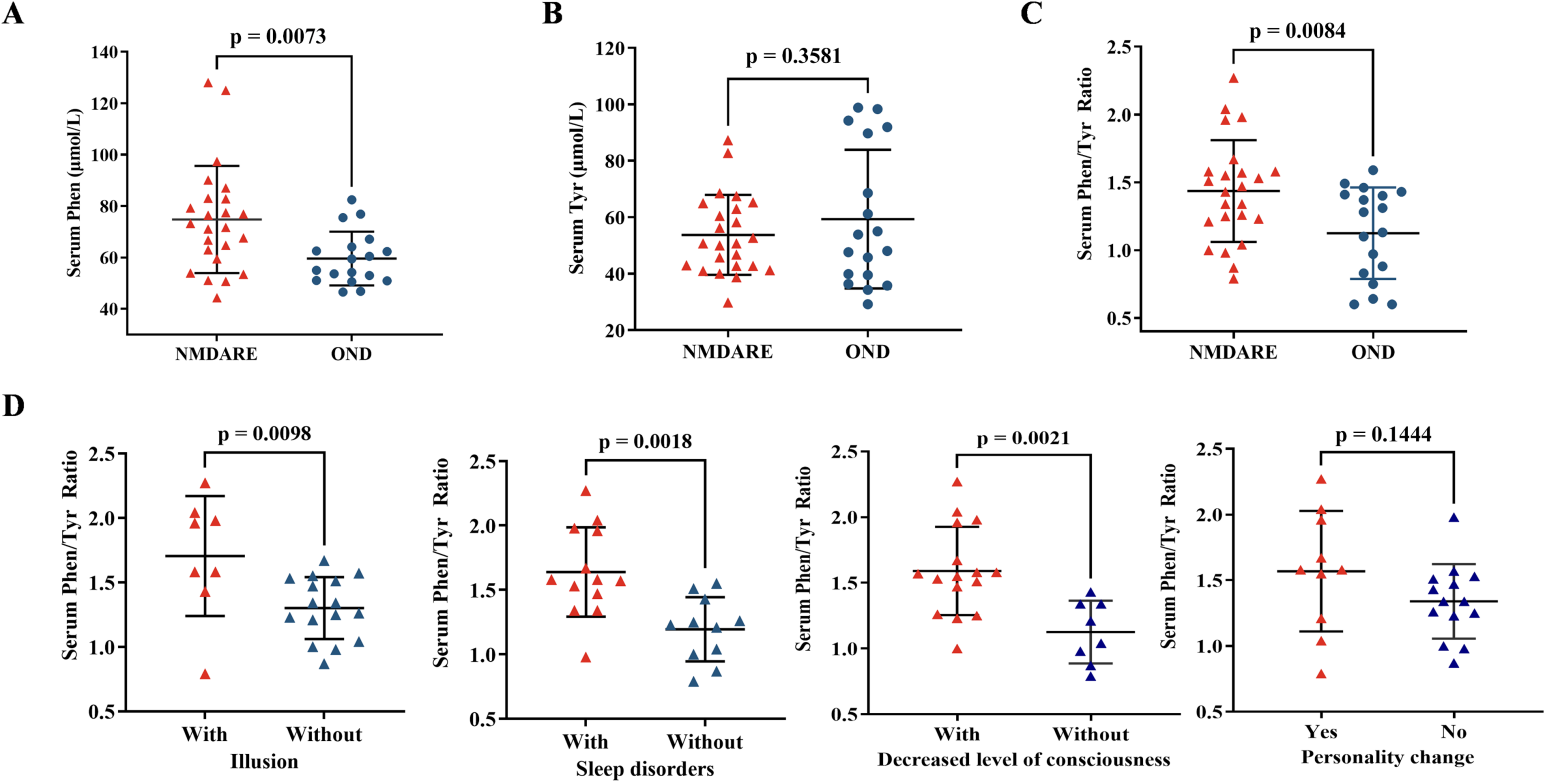
Comparison of serum phenylalanine, serum tyrosine, and serum Phe/Tyr ratio between anti-NMDAR encephalitis (NMDARE) and OND. **(A)** The serum Phenylalanine level was increase in anti-NMDAR encephalitis group, comparing with OND group (NMDARE vs. OND, *p* = 0.0073). **(B)** The serum Tyrosine level had no significant differences in anti-NMDAR encephalitis group, comparing with OND group. **(C)** The serum Phe/Tyr ratio was elevated significantly in anti-NMDAR encephalitis, comparing with OND group (NMDARE vs. OND, *p* = 0.0084). **(D)** The serum Phe/Tyr ratio was higher in anti-NMDAR encephalitis patients with illusion (*p* = 0.0098), sleep disorders (*p* = 0.0018), and decreased level of consciousness (*p* = 0.0021), however, the serum Phe/Tyr ratio had no differences in anti-NMDAR encephalitis patients with personality changes. Differences were assessed by student *t*-test analysis.

As shown in Table 1 and Figure 1A, the level of serum Phe in the anti-NMDAR encephalitis group (74.73 ± 20.85 μmol/L) was significantly higher compared to the OND group (58.86 ± 10.49 μmol/L, *p* = 0.0073). The level of serum tyrosine did not significantly differ between the anti-NMDAR encephalitis group and the OND group (52.95 ± 21.81 μmol/L, *p* > 0.3581, Figure 1B). The serum Phe/Tyr ratio was significantly increased in the anti-NMDAR encephalitis group (1.43 ± 0.38) compared to the OND group (1.12 ± 0.34, *p* = 0.0084, Figure 1C). CSF Phe and Tyr levels were also measured in both groups (Table 1), but statistical analysis showed no significant difference between the groups (data not shown).

Previous studies have established that fluctuations in tyrosine metabolism are frequently associated with behavioral changes (29). Thus, we examined the serum Phe/Tyr ratio in anti-NMDAR encephalitis patients with abnormal behavior or decreased consciousness. Patients with anti-NMDAR encephalitis who exhibited illusions (*p* = 0.0098), sleep disorders (*p* = 0.0018), and decreased consciousness (*p* = 0.0021) had an increased serum Phe/Tyr ratio, while those with personality changes showed no significant differences (Figure 1D).

### 3.3 Serum Phe/Tyr ratio correlated with TNF-*α* levels and clinical parameters

**Figure 2:**
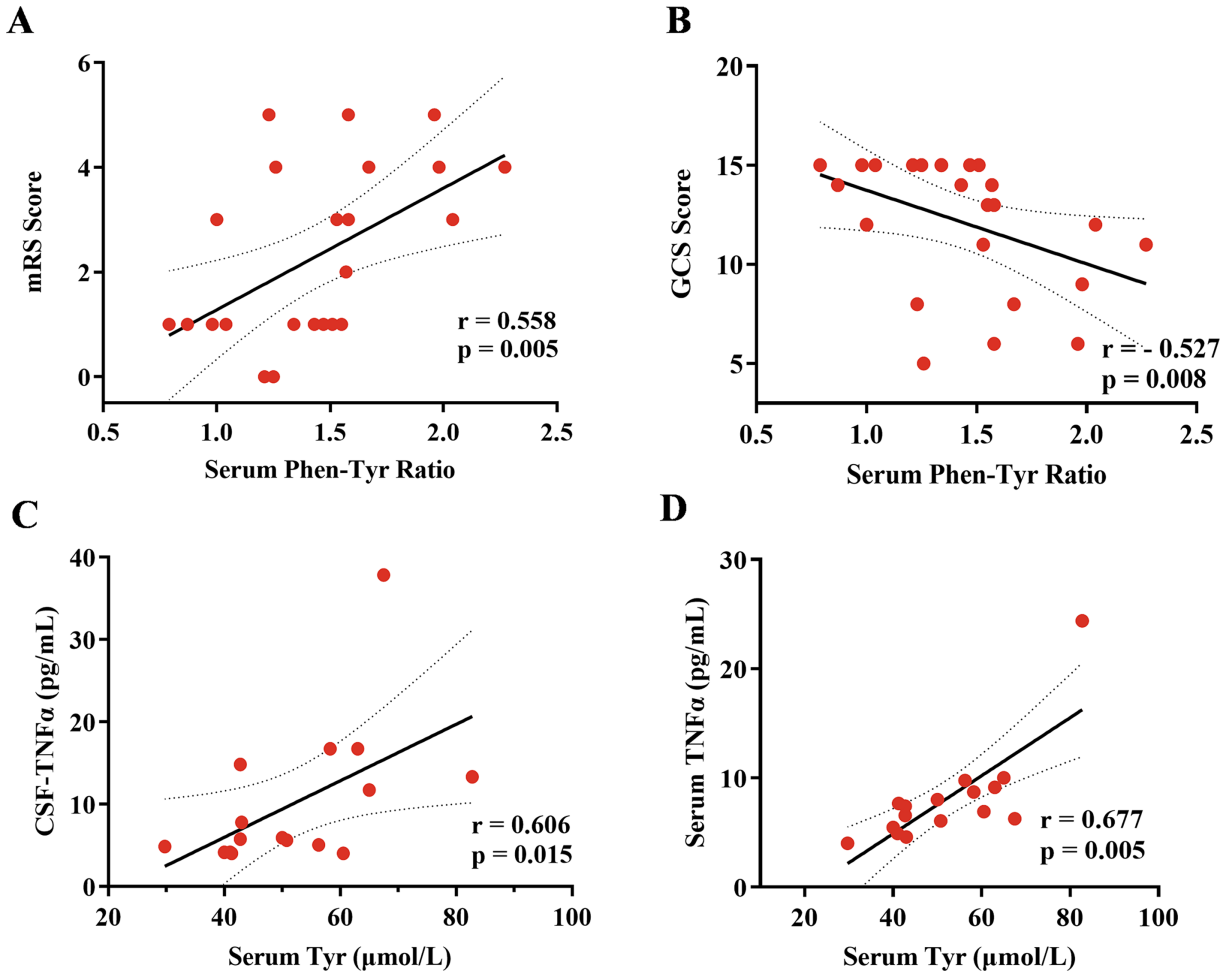
Correlation analysis of serum phenylalanine/tyrosine (Phe/Tyr)ratio with clinical scores and tumor necrosis factor (TNF)-*α* both in CSF and serum. **(A,B)** The serum Phe/Tyr ratio was positively related to the mRS scale (*r* = 0.558, *p* = 0.005) and negatively related to the GCS scores (*r* = −0.527, *p* = 0.008), suggesting that the increase serum Phe/Tyr ratio associated with poor clinical outcomes. **(C,D)** The serum Tyrosine (Tyr) level was positively related to TNF-α both in CSF (*r* = 0.606, *p* = 0.015) and serum (*r* = 0.677, *p* = 0.005) levels. Spearman correlation coefficients were used for the analysis.

The mRS and GCS scores were used to evaluate the severity of clinical outcomes. The serum Phe/Tyr ratio was correlated with the mRS score (Figure 2A, *r* = 0.558, *p* = 0.005) and the GCS score (Figure 2B, *r* = −0.527, *p* = 0.008). A higher serum Phe/Tyr ratio indicated more severe anti-NMDAR encephalitis. Additionally, as a pro-inflammatory cytokine, TNF-*α* levels were significantly elevated in anti-NMDAR encephalitis patients (30). The CSF TNF-α levels (Figure 2C, *r* = 0.606, *p* = 0.015) and serum TNF-α levels (Figure 2D, *r* = 0.677, *p* = 0.005) were both associated with the serum Phe/Tyr ratio.

### 3.4 Serum Phe/Tyr ratio showed a predictive value in clinical outcomes

**Figure 3:**
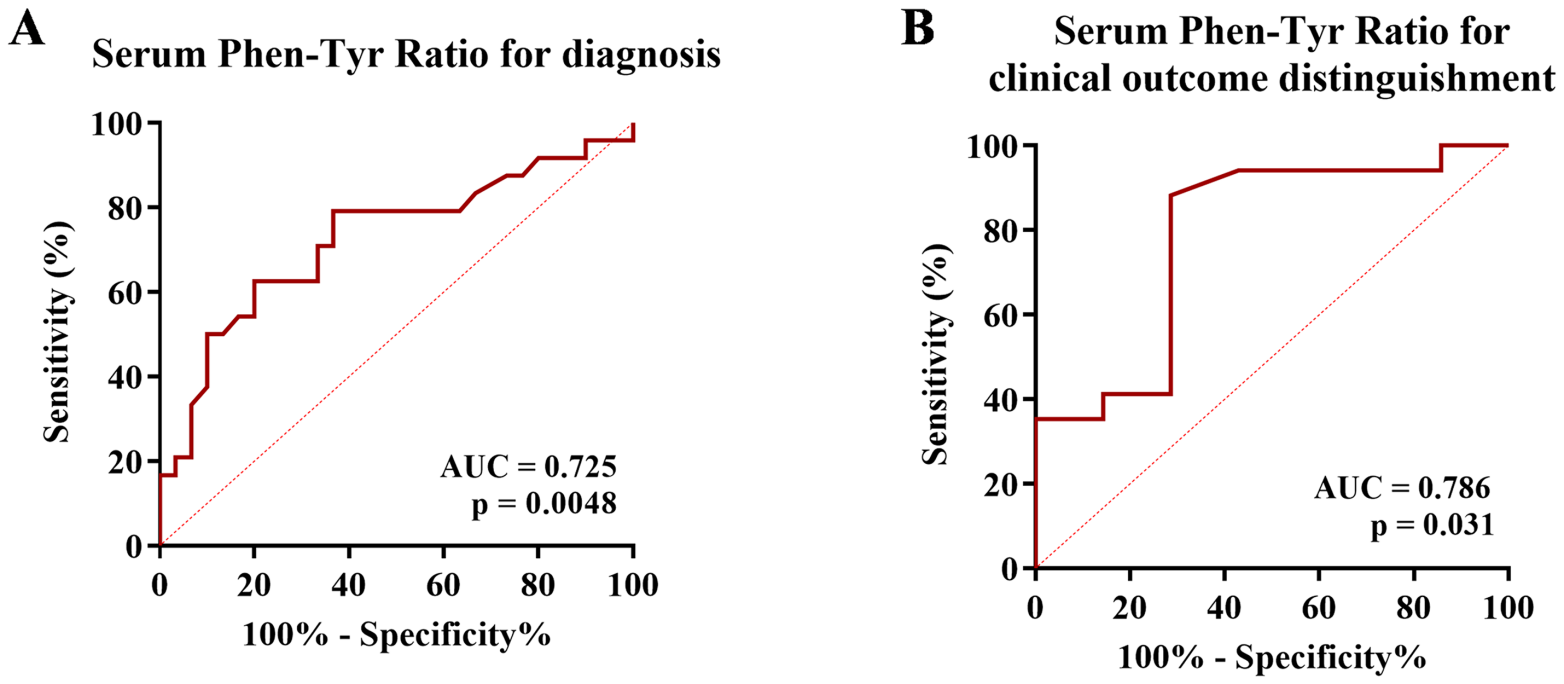
The predictive value of serum Phe/Tyr ratio for anti-NMDAR encephalitis. **(A)** Receiver operating characteristic curve (ROC) analysis was applied to determine the diagnostic value of serum Phe/Tyr ratio (AUC = 0.7250, *p* = 0.0048). **(B)** Clinical outcome of each patient was classified as good or poor according to the mRS score (mRS > 3 poor outcome, mRS ≤ 3 good outcome). ROC analysis for serum Phe/Tyr ratio (AUC = 0.786, *p* = 0.031) in distinguishing good and poor clinical outcomes in anti-NMDAR encephalitis.

We performed ROC analyses to explore the diagnostic value of the serum Phe/Tyr ratio for anti-NMDAR encephalitis. The serum Phe/Tyr ratio effectively differentiated anti-NMDAR patients (*N* = 24) from the OND group (*N* = 18), with an AUC of 0.725 (Figure 3A, *p* = 0.0048).

To further assess the potential of the serum Phe/Tyr ratio in identifying the severity of neurological impairments in anti-NMDAR encephalitis patients, we conducted a receiver operating characteristic (ROC) curve analysis. We classified clinical outcomes using the modified Rankin Scale (mRS) score as the classification cut-off point (mRS score of 3, moderate disability, as the median; a score of >3 indicating poor outcome; a score of ≤3 indicating good outcome). Our findings revealed that the serum Phe/Tyr ratio exhibited predictive capabilities for clinical outcomes in anti-NMDAR encephalitis. The AUC of the serum Phe/Tyr ratio for distinguishing between poor and good outcomes in anti-NMDAR encephalitis patients was 0.786 (*p* = 0.031, Figure 3B). These results indicate that the serum Phe/Tyr ratio holds diagnostic and predictive potential for determining clinical outcomes in anti-NMDAR encephalitis.

## 4 Discussion

This study aims to demonstrate that tyrosine metabolism is altered in anti-NMDAR encephalitis patients with psychiatric symptoms and is related to clinical outcomes. Our results show that: (1) the serum level of phenylalanine increased in anti-NMDAR encephalitis patients compared to OND patients, while serum levels of tyrosine showed no differences; (2) the serum Phe/Tyr ratio was elevated in anti-NMDAR encephalitis patients compared to OND patients; (3) the serum Phe/Tyr ratio was higher in anti-NMDAR encephalitis patients with psychiatric symptoms (including illusions, sleep disorders, and decreased levels of consciousness); (4) the serum Phe/Tyr ratio was related to mRS score, GCS score, and inflammatory molecules (TNF-*α*); and (5) the serum Phe/Tyr ratio was significantly associated with disease severity, suggesting its potential as a biomarker to improve treatment or prevention in clinical practice for anti-NMDAR encephalitis.

Both phenylalanine and tyrosine are indispensable aromatic amino acids essential to our diet (31). Unlike other neurotransmitter pathways, the synthesis and release rates of catecholamines are particularly sensitive to the levels of their amino acid precursors (phenylalanine and tyrosine), which are influenced by their availability in the blood (31). Therefore, the synthesis rates are greatly affected by physiological factors, particularly changes in dietary intake, which influence the levels of these amino acids (31, 32). The functional implications of these changes are significant and provide important insights into the regulation and effects of these crucial neurotransmitters.

Tyr is the preferred substrate for tyrosine hydroxylase in catecholamine synthesis, involving hydroxylation to dihydroxyphenylalanine (DOPA). Recently, Yi Zhang et al. identified a novel microbial levodopa metabolism pathway mediated by tyrosine decarboxylase, primarily encoded by the tyrosine decarboxylase gene, which suggests the potential for drug response prediction (33). **Phe** is related to classic phenylketonuria (PKU), caused by defective activity of phenylalanine hydroxylase that converts phenylalanine to tyrosine, and can affect brain development and function depending on the timing of exposure to elevated levels. The specific mechanisms of phenylalanine-induced brain damage are not completely understood but likely involve the impairment of synaptogenesis (34). These two neurotransmitter precursors were measured in peripheral blood to illustrate the relationship between neurological symptoms and metabolism in many diseases. Lanser et al. found that inflammation-induced phenylalanine accumulation (as reflected by the Phe/Tyr ratio) is related to anemia, fatigue, and depression in cancer (35). Mario et al. investigated that patients with fatigue, sleep disturbance, and neurological symptoms during and after COVID-19 might be associated with inflammation-induced changes in phenylalanine metabolism (36). Katharina et al. also used the Phe/Tyr ratio to reflect catecholamine pathway activity to explain the biological mechanisms linking SARS-CoV-2 infection and mental health (37). These studies provide evidence that the Phe/Tyr ratio, reflecting neurotransmitter metabolism, is associated with inflammation and psychiatric symptoms. In our study, we also found an elevation of serum phenylalanine levels in anti-NMDAR encephalitis (Figure 1A) and an increase in the serum Phe/Tyr ratio in anti-NMDAR encephalitis patients with psychiatric symptoms (Figure 1D). Additionally, we investigated the relationship between serum tyrosine levels and TNF-*α* levels (both CSF and serum), indicating that inflammatory factors may play a role in the altered tyrosine metabolism in anti-NMDAR encephalitis (Figures 2C,D). Those suffering from anti-NMDAR encephalitis, particularly teenagers and adults, frequently present with behavioral changes (such as psychosis, delusions, hallucinations, agitation, aggression, or catatonia) and insomnia, alongside irritability. These symptoms are typically followed by speech dysfunction, dyskinesias, memory deficits, autonomic instability, and a reduced level of consciousness (38, 39). Our results suggest that an increase in the serum Phe/Tyr ratio in patients with anti-NMDAR encephalitis is often associated with the presence of psychiatric symptoms, indicating that phenylalanine and tyrosine metabolism may play a role in the pathobiology of anti-NMDAR encephalitis. To investigate the relationship between clinical outcomes and the serum Phe/Tyr ratio, we applied mRS scales and GCS scores to estimate the severity of clinical characteristics in these patients. We found that the Phe/Tyr ratio has potential value in the diagnosis and prognosis of anti-NMDAR encephalitis and may aid in differentiating it from psychosis. However, it remains unclear whether neuroinflammation affects phenylalanine and tyrosine metabolism in anti-NMDAR encephalitis, which will be the focus of our future research.

Several limitations in our study should be acknowledged. First, as a single-center study, biases related to limited sample size and varying follow-up periods might exist. Second, further exploration is needed to understand the pathways through which phenylalanine contributes to the psychotic symptoms of anti-NMDAR encephalitis. Additionally, we can examine the correlation between serum levels of Phe and Tyr with disease severity, imaging features, and prognosis. In our study, the statistical power of the serum Phe level was 0.472. The statistical power of the serum Phe/Tyr level was 0.707. Thus, although we found that Phe was elevated in anti-NMDA encephalitis, our results were limited by the sample size. This differential diagnostic value in anti-NMDAR encephalitis needs to be further explored.

In this study, we aimed to explore the relationship between tyrosine metabolism and psychiatric symptoms in anti-NMDAR encephalitis. Using LC–MS to measure serum levels of phenylalanine and tyrosine, we found an increase in serum phenylalanine levels in patients with anti-NMDAR encephalitis. Our extensive research revealed that the phenylalanine-tyrosine ratio in anti-NMDAR encephalitis patients with psychiatric symptoms is significantly increased and correlated with clinical outcomes. Specifically, our results suggest that tyrosine metabolism is altered in anti-NMDAR encephalitis and is related to disease severity, highlighting the serum Phe/Tyr ratio as a potential biomarker.

## 5 Conclusion

Our study is the first to report that neurotransmitter precursors (phenylalanine, tyrosine) are related to psychiatric symptoms in anti-NMDAR encephalitis. We demonstrated that the serum Phe/Tyr ratio is increased in patients with psychiatric symptoms and has the potential to reflect clinical outcomes in anti-NMDAR encephalitis, suggesting the serum Phe/Tyr ratio as a diagnostic and prognostic biomarker.

## Data Availability

The raw data supporting the conclusions of this article will be made available by the authors, without undue reservation.
